# Circulating Copper Is Associated with Inflammatory Biomarkers in Greek Older Adults with Osteoarthritis

**DOI:** 10.1007/s12011-023-03801-1

**Published:** 2023-08-22

**Authors:** Charalampia Amerikanou, Evdokia Valsamidou, Sotirios Karavoltsos, Dimitra Tagkouli, Aikaterini Sakellari, Maria Kontou, Dimitra Houhoula, Nick Kalogeropoulos, Panagiotis Zoumpoulakis, Andriana C. Kaliora

**Affiliations:** 1 Department of Nutrition and Dietetics, School of Health Science and Education, Harokopio University of Athens, 70 El. Venizelou Ave, 17676 Athens, Greece; 2 Department of Chemistry, National and Kapodistrian University of Athens, Panepistimiopolis Zografou, 15784 Athens, Greece; 3 TheraCell Advanced Biotechnologies, 14564 Kifissia, Greece; 4 Department of Food Science and Technology, University of West Attica, Ag. Spyridonos, 12243 Egaleo, Athens, Greece

**Keywords:** Osteoarthritis, Circulating metals/metalloids, MicroRNAs, Inflammatοry markers, Copper, Lifestyle

## Abstract

Osteoarthritis (OA) is the most common form of arthritis, that causes a significant decrease in the quality of life of the afflicted and constitutes a great burden for the socioeconomic system. Trace elements and heavy metals are implicated in the pathophysiology of OA, exacerbating inflammatory and oxidative stress responses. The aim of this cross-sectional study was to quantify metals in plasma samples of Greek OA patients and explore their link with disease related parameters, health status or quality of life, as well as epigenetic OA markers. This is the first study on plasma metal levels in Greek knee OA patients. To achieve precision in plasma metal and miRNA measurements, high-quality samples were selected from a subset of 34 participants (NCT04783792). Demographic, quality of life, clinical, biochemical, inflammation, oxidative stress, and anthropometric parameters, as well as microRNA levels were assessed. Significant correlations were found between circulating metals with OA related parameters or with measured microRNAs. Also, significant positive associations between plasma copper (Cu) levels and CRP (*p* = 0.033) or IL-6 (*p* = 0.001) occurred when adjusting for age, gender, BMI, physical activity level, smoking, disease severity, total arthroplasty, and dietary intake of the respective metal. Cu’s role in OA is bidirectional, and this study confirms the findings that in OA, Cu is positively associated with inflammation. Such relationships between lifestyle, environment and OA enhance our understanding and encourage further study on metals related to OA inflammation.

## Introduction

Osteoarthritis (OA) is a non-communicable, degenerative disease of the articular cartilage of the joints. It is a heterogeneous and multifaceted malady with genetic, mechanical, and environmental factors contributing to the deterioration of the consistency of the articular cartilage [1]. The onset of the disease can occur as early as the 30s; however, its prevalence increases exponentially with age, particularly in the female gender [2]. OA is the most common form of arthritis globally with serious person-level repercussions such as pain and decrease in functionality and in quality of life (QoL) of the afflicted, and society-level burdens in the socioeconomic realm [3]. Treatments can only offer palliative relief and chronic pharmacological approaches have serious side effects. Non-pharmacological options include physical therapy, weight management, incorporation of appropriate physical activities, education of patients and supplements to reinforce health and manage symptoms [4], as well as the use of nutraceuticals [5]. The pathophysiology of OA is complex, and it affects all the tissues of the joint, leading to inflammation of the synovium, the formation of bone spurs and the degradation of the articular cartilage; sustained chronic low-grade inflammation and oxidative stress (OS) generate metabolites that further aggravate the progression of the disease through positive feedback loops [6].

Metals have been found to be implicated in biological processes such as inflammation and OS. They can be either beneficial or harmful depending on the tissue, duration of exposure, type and levels of metals. Several metals and metalloids (i.e., aluminum (Al), mercury (Hg), arsenic (As), cadmium (Cd), chromium (Cr), copper (Cu)) occur naturally in Earth’s crust [7]. Toxic metals/metalloids such as Hg, Cr, Cd, As, and lead (Pb) may provoke deleterious effects on human metabolism and mechanistically their toxicity can be attributed to induction of OS and depletion of antioxidant capacities [8]. On the contrary, trace or essential metals (i.e., sodium (Na), potassium (K), magnesium (Mg), and calcium (Ca)) are necessary for human body at specific concentrations [9]. Their biological roles include among others catalytic actions, stabilization of oxidation states, and structural functions [9], with their ligand binding strengths and mobilities being the most significant [10]. Given that metals play a pivotal role in metabolic functions which are altered in OA [11], the interplay between circulating metals and OA including the inflammatory mediators of the disease is of particular interest.

MicroRNAs (miRNAs) are endogenous single-stranded, noncoding sequences of 20–25 nucleotides with their biological role being highlighted as negative regulators of gene expression at post-transcriptional level [12]. Due to their regulatory role, miRNAs have recently gained considerable attention regarding their involvement in OA pathophysiology. In OA they participate in the homeostasis of chondrocytes and cartilage and in vivo modulation of them has been shown to affect the progression of the disease [13]. What is more, several studies have investigated the associations between metals and miRNAs in signaling pathways and in disease development [14–17]. However, none of them has addressed whether miRNA regulation mediates the role of metals in OA or whether metal exposure influences OA through epigenetic miRNA alterations.

On that aspect, the aim of our study was to quantify plasma metals in Greek OA patients and explore their associations with disease related parameters. Additionally, because it has been hypothesized that specific miRNAs are dysregulated in OA, their quantification herein was conducted to provide evidence of a potential mediating role. Exploring such relationships among lifestyle, environment and development of the disease broadens our understanding in disease pathophysiology, management and/or prevention.

## Methods

### Ethics

This is a cross-sectional study that used baseline data from a subset of patients from a randomized controlled trial (RCT) that evaluated a standardized nutraceutical supplement in pain, quality of life, inflammation and OS in OA (manuscript in peern review process). The RCT was approved by Harokopio University Ethics Committee (13/21–2-2020) and Evgenidio Hospital Scientific Board (29/19–02-2019) and was registered in the ClinicalTrials.gov database with the identifier NCT04783792. The protocol followed all the principles of the Declaration of Helsinki 2013 and the Data Protection Act 2018 and all patients gave their informed consent before entering the study. More details on the clinical trial study design and sample size calculation are given elsewhere (manuscript in peer review process).

### Participants

Individuals above the age of 35 with unilateral or bilateral OA of the knee confirmed by radiography (stage II, III, or IV according to Kellgren and Lawrence classification, K&L) [18] and by the American College of Rheumatology (ACR) clinical and radiographic criteria for OA [19] were enrolled. To confirm that radiographic findings correlated with the symptoms, patients had to have at least moderate symptoms of pain in the target knee depicted as intermittent (pain during the last 7 days) or constant pain above 4 in the Western Ontario and McMaster Universities Osteroarthritis (WOMAC) pain subscale and in visual analogue scale (VAS). In bilateral OA, the target knee was determined by the highest VAS pain score. Volunteers had to be able to walk without support devices such as a walking stick, crutches, or a kneecap. Main exclusion criteria were patients with OA who were having physical therapy or transcutaneous electrical nerve stimulation prior the trial, rheumatoid arthritis, fibromyalgia, spinal disorders or any other musculoskeletal disorders that according to the physician were a bias (i.e., chronic low back pain), a scheduled knee surgery or any other programmed surgery during the trial, those using injected or per-os corticosteroids within 2 months prior to randomization and during the trial or those with adaptations to diet/food supplements 1 month prior or during the recruitment and throughout the trial. Patients were advised to adhere to their lifestyle habits prior the randomization to the trial, and upon discomfort due to pain they were allowed to use rescue medication (paracetamol and non-steroidal anti-inflammatory drugs).

### Baseline Assessments

At baseline the medical history of the patients was recorded along with information regarding the progress of OA and related surgeries such as total knee arthroplasty (TKA), demographics, and smoking. Furthermore, anthropometric measurements were taken that included height (cm) and body weight (kg) to compute body mass index (BMI) as weight (kg)/height (m)^2^, as well as waist circumference (cm) and hip circumference (cm) to compute the waist-hip ratio (WHR). Finally, validated questionnaires for OA that evaluate core symptoms of the disease and physical function were filled out. Those included (1) VAS for pain that is a numeric continuum from 0 (no pain) to 100 mm (worst pain) and recorded the worst feeling of pain patients had on the target knee during the last 7 days, (2) WOMAC that consists of 24 items that are divided into 3 subscales; the subscale of pain (5 items): during walking, using stairs, in bed, sitting or lying, and standing upright, the subscale of stiffness (2 items): after first waking and later in the day and the subscale of physical function (17 items): using stairs, rising from sitting, standing, bending, walking, getting in/out of a car, shopping, putting on/taking off socks, rising from bed, lying in bed, getting in/out of bath, sitting, getting on/off toilet, heavy domestic duties, light domestic duties The 24 test questions are scored on a scale of 0–4, which correspond to the following: none (0), mild (1), moderate (2), severe (3), and extreme (4). The scores for each subscale are summed up; with a possible score range of 0–20 for pain, 0–8 for stiffness, and 0–68 for physical function that together compute a percentage of the WOMAC score. Higher scores indicate worse pain, stiffness, and functional limitations [20].

Regarding QoL, ShortForm36 (SF-36) was used. This is a health-related questionnaire with 36 questions about physical functioning, role limitations due to physical health, due to emotional problems, energy/fatigue, emotional well-being, social functioning, bodily pain, and general health. All eight dimensions of the questionnaire were computed via an online tool [21] and higher scores indicated a better health status. Physical activity was assessed via the International Physical Activity Questionnaire Short Form (IPAQ-SF) and expressed as metabolic equivalent task in minutes per week (MET-min/week) [22]. Adherence to the Mediterranean diet was evaluated using the Mediterranean Diet score (MedDiet score) [23]. The total score of MedDiet ranges from 0 to 55 with higher values indicating greater adherence to the Mediterranean diet. Also, 24-h recalls were used to assess metals dietary intake using Nutritionist Pro™ software package (Axxya Systems, Stafford, TX, USA).

### Blood Collection

Blood sample collection (20 mL) was performed in the subjects after overnight fasting. For plasma and serum isolation whole blood was collected in blood tubes. The vacutainers were centrifuged for 10 min at a speed of 3000 rpm in order to receive the supernatant. Plasma and serum were stored in Eppendorf tubes in − 80 °C until further analysis.

For accuracy in miRNA and metals quantification, samples used were not hemolyzed and were of adequate volume for the Inductively Coupled Plasma Mass Spectometry (ICP-MS) analysis or of high purity extracted RNA for the miRNA quantification. These characteristics were met in 34 out of 60 samples of the RCT.

### Analysis of Serum Biomarkers

Biochemical analysis was performed in serum with an automatic biochemical analyzer (Cobas 8000 analyzer, Roche Diagnostics GmbH, Mannheim, Germany) and included vitamin D, high-density lipoprotein cholesterol (HDL), triglycerides (TG), glucose, urea, C-reactive protein (CRP), albumin, creatinine, serum glutamic oxaloacetic transaminase (SGOT), and serum glutamic pyruvic transaminase (SGPT). Inflammation and OS markers that were measured in duplicate in serum through sandwich enzyme-linked immunosorbent assay (ELISA) included: interleukin-6 (IL-6) (R&D Systems, Inc. Minneapolis, MN, USA), tumor necrosis factor-alpha (TNF-α) (Thermo Fisher Scientific Inc., Waltham, MA, USA), myeloperoxidase (MPO) (Thermo Fisher Scientific Inc., Waltham, MA, USA) and oxidized low-density lipoprotein (oxLDL) (Mercodia, AB, Uppsala, Sweden).

### MicroRNA Quantification in Plasma

Four miRNAs (mir21-5p, mir126-3p, mir146a-5p, mir155-5p) were quantified in plasma of patients with knee OA through reverse transcription quantitative real-time PCR (RT-qPCR) analysis. The above miRNAs were selected after thorough bibliographic search as they have been reported to mediate the development, pathogenesis and progression of OA. MagMAX™ mirVana™ Total RNA Isolation Kit (Thermo Fisher Scientific Inc., Waltham, MA, USA) was used for the extraction of total RNA (enriched in miRNAs) from plasma according to the manufacturer’s protocol. RNA quality and quantity was assessed using an Implen P330 nanophotometer (Implen GmbH). cDNA was produced using TaqMan® Advanced miRNA cDNA Synthesis Kit (Thermo Fisher Scientific Inc., Waltham, MA, USA). MiRNA expression was determined in StepOnePlus™ Real-Time PCR System (Thermo Fisher Scientific Inc., Waltham, MA, USA) in duplicates using TaqMan® Advanced miRNA Assays (for I-miR-21-5p (assay ID #477975_mir) fIhsa-miR-126-3p (assay ID #477887_mir)Ior hsa-miR-146a-5p (assay ID #478399_mir)Id for hsa-miR-155-5p (assay ID #477927_mir). ExpressionSuite™ Software was used to calculate relative expression using the comparative Ct (ΔΔCt) method and an exogenous control (Caenorhabditis elegans miRNA Cel-miR-39-3p (assay ID #478293_mir)) was used for normalization. Relative levels of miRNAs were compared to a reference sample, and results are presented as fold change using the 2^−ΔΔCt^ formula, where ΔΔCt = [(Ct gene of interest-Ct control) sample] – [(Ct gene of interest-Ct control) reference sample].

### Inductively Coupled Plasma Mass Spectrometry for Metal Quantification

Plastic materials contacting blood samples were thoroughly washed, subsequently soaked in dilute HNO_3_ (Merck, Darmstadt, Germany), and finally rinsed with ultrapure water of 18.2 MΩ cm (Millipore, Bedford, MA, USA). Samples dilution was performed with micropipettes subject to regular calibration. Class A volumetric glassware was used in order to prepare all required solutions. For samples digestion a mixture of HNO_3_ (suprapur 65%) (Merck) and H_2_O_2_ (suprapur 30%) (Merck) was used and the procedure followed is described by Jin et al. [24] and Batáriová et al. [25], modified to a small extent. Analysis of the digested samples was carried out employing an ICP-MS (Thermo Scientific ICAP Qc, Waltham, MA USA), with measurements performed in a single collision cell mode, with kinetic energy discrimination (KED) using pure He. Internal standards (^45^Sc, ^103^Rh) were used for the correction of matrix induced signal suppressions and instrumental drift. Each sample was analyzed twice, with the average of the two measurements being used for the statistical treatment of data. The limits of detection (LOD), calculated according to US EPA [26], varied from 0.03 μg L^−1^ for Cs and Tl to 0.8 for Fe. For statistical calculations, values below the LOD were assigned the method detection limit divided by √2.

### Statistical Analysis

The Shapiro–Wilk test was used to assess normal distribution. Continuous variables are presented as medians (interquartile range, IQR) or as mean ± standard deviation (SD). Categorical data are presented as counts and percentages. Student’s *t*-test (normally distributed data) and Mann–Whitney *U* test (non-normally distributed data) were used for the comparisons of means between two independent groups. Correlations between two variables were investigated using Spearman’s correlation test. Linear models were used to test the associations of metals with study parameters that showed a significant bivariate correlation. Not normally distributed parameters were log transformed where needed. Statistical analysis was performed using SPSS 21.0 (IBM, SPSS Inc., Chicago, IL, USA).

## Results

Since accurate plasma metal and miRNA measurements require high-quality samples, a subset of 34 participants (out of 60 participants) were selected to be included in this study. The descriptive characteristics of the study population are presented in Table 1. OA patients had a mean age of 60.2 ± 10.1, and a mean BMI over 30 (30.1 ± 5.0). They were mostly females, married or divorced, with moderate to severe OA.

**Table 1:** Descriptive characteristics of the sample

		OA patients (*N* = 34) Mean ± SD or median (IQR)
Demographic/anthropometric characteristics		
Age (years)		60.2 ± 10.1
Sex	Females, *N* (%)	21 (61.8)
	Males, *N* (%)	13 (38.2)
BMI (kg/m^2^)		30.1 ± 5.0
WC (cm)		103.9 ± 10.9
WHR		0.9 ± 0.1
Smoking	Yes, *N* (%)	7 (20.6)
	No, *N* (%)	27 (79.4)
Marital status	Married/divorced, *N* (%)	28 (82.4)
	Unmarried, *N* (%)	6 (17.6)
Education	1–9 years, *N* (%)	7 (20.6)
	10–12 years, *N* (%)	6 (17.6)
	> 12 years, *N* (%)	21 (61.8)
Disease indices		
Kelgren and Lawrence	2, *N* (%)	6 (17.6)
	3,* N* (%)	20 (58.9)
	4, *N* (%)	8 (23.5)
VAS		7.0 ± 1.6
WOMAC pain		7.0 ± 3.6
WOMAC stiffness		2.0 (2.5)
WOMAC function		14.0 (17.5)
WOMAC total score (%)		21.9 (24.4)
Biochemical parameters		
Vitamin D (ng/mL)		26.2 ± 12.8
TC (mg/dL)		200.0 ± 37.8
LDL (mg/dL)		118.4 ± 34.3
HDL (mg/dL)		53.2 ± 12.0
TG (mg/dL)		123.0 (74.0)
Glucose (mg/dL)		95.0 (13.5)
Creatinine (mg/dL)		0.7 (0.3)
Urea (mg/dL)		34.5 ± 11.5
Albumin (g/dL)		4.4 ± 0.3
SGOT (U/L)		19.0 (5.0)
SGPT (U/L)		18.0 (14.0)
Inflammatory/oxidative stress biomarkers		
CRP (mg/L)		2.8 (2.5)
IL-6 (pg/mL)		1.9 (1.2)
TNFa (pg/mL)		0.9 (0.6)
OxLDL (U/L)		66.7 (40.8)
MPO (ng/mL)		56.4 (61.9)
Relative expression of OA-related miRNAs (RQ)		
miR-21		0.752 (1.480)
miR-126		0.360 (0.730)
miR-146a		0.640 (1.670)
mir-155		1.922 (7.100)
QoL indices		
SF-36 (physical functioning)		53.3 ± 24.3
SF-36 (bodily pain)		49.9 ± 45.0
SF-36 (energy/fatigue)		56.5 ± 21.0
SF-36 (role limitations due to emotional problems)		100.0 (66.7)
SF-36 (Role limitations due to physical health)		50.0 (100.0)
SF-36 (emotional well-being)		63.8 ± 18.4
SF-36 (social functioning)		75.0 (62.5)
SF-36 (general health)		61.5 (20.2)
PAL (MET-min/week)		1267.0 (1596.0)
MedDiet score		30.2 ± 3.9

Continuous variables are presented as medians (interquartile range) or as mean ± standard deviation (SD). Categorical data are presented as counts and percentages

*OA* osteoarthritis, *BMI* body mass index, *WC* waist circumference, *WHR* waist to hip ratio, *VAS* visual analogue scale, *WOMAC* Western Ontario and McMaster Universities Osteoarthritis Index, *TC* total cholesterol, *LDL* low density lipoprotein, *HDL* high density lipoprotein, *TG* triglycerides, *SGOT* serum glutamic-oxaloacetic transaminase, *SGPT* serum glutamic-pyruvic transaminase, *CRP* C-reactive protein,, *IL-6* interleukin-6, *TNF-α* tumor necrosis factor-alpha, *oxLDL* oxidized low-density lipoprotein, *MPO* myeloperoxidase, *RQ* relative quantification, *QoL* quality of life, *SF-36* short form-36, *PAL* physical activity level as assessed by International Physical Activity Questionnaire Short Form

In Table 2, plasma levels of metals in all participants and in males or females are presented. Distribution between sexes was similar in all metals examined.

**Table 2:** Plasma metals in all participants (total), males and females

	Total (*N* = 34)	Males (*N* = 13)	Females (*N* = 21)	*P*
V (μg/L)	0.36 (1.17)	0.35 (1.27)	0.26 (1.23)	0.753
Cr (μg/L)	1.59 (4.72)	0.07 (5.02)	0.64 (4.57)	0.753
Mn (μg/L)	1.85 (5.68)	2.98 (9.98)	1.22 (4.4)	0.181
Fe (μg/L)	1348 (413)	1371 (594)	1332 (492)	0.727
Co (μg/L)	0.35 (0.72)	0.24 (1.07)	0.49 (0.61)	0.529
Ni (μg/L)	3.65 (2.54)	3.69 (6.54)	4.53 (2.81)	0.889
Cu (μg/L)	1119 ± 186	1057 ± 155	1157 ± 197	0.131
Zn (μg/L)	966 (422)	972 (435)	869 (353)	0.624
As (μg/L)	1.00 (2.11)	1.21 (2.32)	0.30 (1.83)	0.193
Se (μg/L)	52.4 ± 20.7	51.7 ± 21.9	52.8 ± 20.4	0.887
Rb (μg/L)	387 ± 80	414 ± 72	371 ± 83	0.130
Sr (μg/L)	29.1 ± 9.5	30.8 ± 8.8	28.1 ± 10.0	0.433
Cd (μg/L)	0.06 (0.20)	0.03 (0.21)	0.02 (0.08)	0.400
Cs (μg/L)	0.78 (0.36)	0.80 (0.44)	0.75 (0.32)	0.309
Ba (μg/L)	5.67 (8.87)	5.20 (8.97)	6.14 (7.15)	0.807
Tl (μg/L)	0.05 (0.11)	0.06 (0.35)	0.05 (0.08)	0.529
Cu/Zn ratio	1.2 (0.5)	1.2 (0.6)	1.2 (0.5)	0.441

Continuous variables are presented as medians (interquartile range) or as mean ± standard deviation (SD). Categorical data are presented as counts and percentages. Student’s *t*-test (normally distributed data) and Mann–Whitney *U* test (non-normally distributed data) were used for the comparisons of means between two independent groups

*V* vanadium, *Cr* chromium, *Mn* manganese, *Fe* iron, *Co* cobalt, *Ni* nickel, *Cu* copper, *Zn* zinc, *As* arsenic, *Se* selenium, *Rb* rubidium, *Sr* strontium, *Cd* cadmium, *Cs* caesium, *Ba* barium, *Tl* thallium

In Table 3, significant correlations between plasma metals, and all parameters tested related to the disease are presented. Mn was negatively correlated with HDL, Fe with urea, As with VitD and Co/Zn ratio with WHR. Co correlated with miR-21 and mir-146a, Ni with oxLDL, Ba with WOMAC total score, Cu correlated positively with WOMAC stiffness, CRP, IL-6 and TNF-α. A negative correlation was found between Zn and HDL and mir-155. Rb correlated positively with creatinine and inversely with glucose. Some interesting correlations were observed between SF-36 questionnaire dimensions and metals. More specifically, SF-36 (role limitations due to emotional problems) correlated with Cd and Cs, SF-36 (role limitations due to physical health) with Ba and SF-36 (social functioning) with Cs. Finally, SF-36 (emotional well-being) was positively corellated with Cd, Cs and Tl and SF-36 (general health) was positively correlated with Cd, Cs and Cu/Zn.

**Table 3:** Significant correlations among metals/metalloids and parameters reflecting the disease

Correlations	Rho	*P*
WHR with Cu/Zn	− 0.384	0.033
WOMAC stiffness with Cu	0.365	0.034
WOMAC total score (%) with Ba	0.343	0.047
Vit D with As	− 0.378	0.033
HDL (mg/dL) with Mn	− 0.361	0.036
HDL (mg/dL) with Zn	− 0.470	0.005
Glucose (mg/dL) with Rb	− 0.427	0.012
Creatinine (mg/dL) with Rb	0.390	0.022
Urea (mg/dL) with Fe	− 0.341	0.049
CRP (mg/L) with Cu	0.455	0.007
IL-6 (pg/mL) with Cu	0.606	< 0.001
TNF-a (pg/mL) with Cu	0.364	0.035
OxLDL (U/L) with Ni	0.410	0.016
miR-21 with Co	0.398	0.036
miR-146a with Co	0.442	0.021
mir-155 with Zn	− 0.513	0.030
SF-36 (role limitations due to emotional problems) with Cd	0.361	0.039
SF-36 (role limitations due to emotional problems) with Cs	0.405	0.019
SF-36 (role limitations due to physical health) with Ba	0.360	0.040
SF-36 (emotional well-being) with Cd	0.357	0.041
SF-36 (emotional well-being) with Cs	0.345	0.050
SF-36 (emotional well-being) with TI	0.496	0.003
SF-36 (social functioning) with Cs	0.441	0.010
SF-36 (general health) with Cd	0.482	0.004
SF-36 (general health) with Cs	0.471	0.006
SF-36 (general health) with Cu/Zn	0.523	0.002

Correlation analysis was performed with Spearman’s rank correlation. Level of significance was set as 0.05

*WHR* waist to hip ratio, *HDL* high-density lipoprotein, *CRP* C-reactive protein, *IL-6* interleukin-6, *TNF-α* tumor necrosis factor-alpha, *oxLDL* oxidized low-density lipoprotein, *SF-36* short form-36. *Mn* manganese, *Fe* iron, *Co* cobalt, *Ni* nickel, *Cu* copper, *Zn* zinc, *As* arsenic, *Se* selenium, *Rb* rubidium, *Cd* cadmium, *Cs* caesium, *Ba* barium, *Tl* thallium

Linear regression models were applied to examine the associations of the above studied parameters showing significant correlations (Table 4). The first model applied was adjusted for disease severity (as assessed by K&L), the second one for disease severity, age, gender, and BMI while the third one for disease severity, age, gender, BMI, physical activity level, smoking, TKA and dietary intake of the respective metal. Only significant associations in all three models are presented. CRP and IL-6 exhibited a significant positive association with Cu (*p* = 0.033 and *p* = 0.001 respectively in the third model), and WHR was negatively associated with Cu/Zn (*p* = 0.022 in the third model).

**Table 4:** Significant associations between OA-related parameters and metals

	Model 1				Model 2				Model 3			
	CRP											
	Beta ± SE	*P*	*R*	Adj. *R*^2^	Beta ± SE	*P*	*R*	Adj. *R*^2^	Beta ± SE	*P*	*R*	Adj. *R*^2^
Cu (μg/L)	1.002 ± 1.000	**0.008**	0.402	0.108	1.002 ± 1.000	**0.008**	0.494	0.109	1.002 ± 1.000	**0.033**	0.523	0.001
Disease activity^a^	1.161 ± 1.256	0.512			1.169 ± 1.268	0.517			1.135 ± 1.312	0.646		
Age (years)					0.995 ± 1.016	0.755			0.984 ± 1.021	0.496		
Sex (males vs females)					0.767 ± 1.409	0.445			0.851 ± 1.476	0.685		
BMI (kg/m^2^)					0.993 ± 1.033	0.861			0.991 ± 1.040	0.807		
PAL (MET-min/week)									1.000 ± 1.000	0.728		
Smoking status^b^									1.247 ± 1.559	0.624		
TKA (no vs yes)									2.291 ± 2.460	0.728		
Cu dietary intake (mg)									0.832 ± 1.380	0.573		
	IL-6											
	Beta ± SE	*P*	*R*	Adj. *R*^2^	Beta ± SE	*P*	*R*	Adj. *R*^2^	Beta ± SE	*P*	*R*	Adj. *R*^2^
Cu (μg/L)	1.002 ± 1.000	** < 0.001**	0.614	0.336	1.002 ± 1.000	**0.001**	0.711	0.417	1.002 ± 1.000	**0.001**	0.806	0.518
Disease activity	0.977 ± 1.122	0.845			0.951 ± 1.117	0.646			0.845 ± 1.114	0.131		
Age (years)					1.009 ± 1.007	0.263			1.002 ± 1.009	0.845		
Sex (males vs females)					0.734 ± 1.169	0.061			0.782 ± 1.167	0.121		
BMI (kg/m^2^)					1.033 ± 1.016	**0.036**			1.019 ± 1.016	0.257		
PAL (MET-min/week)									0.937 ± 1.000	0.107		
Smoking status									1.675 ± 1.191	**0.007**		
TKA (no vs yes)									1.227 ± 1.425	0.571		
Cu dietary intake (mg)									0.869 ± 1.135	0.281		
	WHR*											
	Beta ± SE	*P*	*R*	Adj. *R*^2^	Beta ± SE	*P*	*R*	Adj. *R*^2^	Beta ± SE	*P*	*R*	Adj. *R*^2^
Cu/Zn	− 0.024 ± 0.010	**0.021**	0.499	0.195	− 0.020 ± 0.008	**0.023**	0.728	0.457	− 0.021 ± 0.009	**0.022**	0.781	0.442
Disease activity	0.026 ± 0.017	0.135			0.025 ± 0.014	0.092			0.017 ± 0.016	0.280		
Age (years)					0.002 ± 0.001	0.041			0.001 ± 0.001	0.309		
Sex (males vs females)					− 0.077 ± 0.020	**0.001**			− 0.071 ± 0.021	**0.001**		
PAL (MET-min/week)									− 1.020 × 10^−5^ ± 0.00	0.061		
Smoking status									0.008 ± 0.025	0.741		
TKA (no vs yes)									0.001 ± 0.050	0.980		
Cu dietary intake (mg)									− 0.006 ± 0.021	0.784		
Zn dietary intake (mg)									0.003 ± 0.002	0.847		

Model 1: adj. for disease activity (as assessed by Kelgren and Lawrence); model 2: adj. for disease activity, age, gender, and BMI; model 3: adj. for disease activity, age, gender, BMI, PAL, smoking, TKA, and dietary intake of the respective metal. CRP and IL-6 were logarithmically transformed during the analysis; therefore, the results (beta ± SE) are presented as exponential, meaning that for every 1-unit increase in the independent variable, the dependent variable changes by the beta coefficient as a percentage. WHR was not logarithmically transformed, meaning that for every 1-unit increase in the independent variable, the dependent variable will change (increase or decrease) by the beta coefficient value

*BMI* body mass index, *CRP* C-reactive protein, *Cu* copper, *IL-6* interleukin-6, *PAL* physical activity level as assessed by International Physical Activity Questionnaire Short Form, *TKA* total knee arthroplasty, *Zn* zinc, *SE* standard error, *Adj. R*^*2*^ adjusted *R*.^2^

^a^Disease activity was assessed by Kelgren and Lawrence

^b^Smoking status as non-smokers vs smokers

*BMI adjustment was not used in these models. Values resulted from linear regression models. Level of significance was set as 0.05. Significant *p* is in bold

## Discussion

The role of metals in human health and development, and progress of diseases is well documented. During the last decades, human exposure to heavy metals has increased due to industrial and everyday life and has been associated with several acute and chronic toxic effects contributing to gastrointestinal, nervous, immune system dysfunction, cancers, and other disorders [27]. On the contrary, trace elements are essential for several biological functions, serving as catalysts in enzyme systems, oxidation reactions, and oxygen transport, whereas their insufficient or excessive concentrations are associated with several diseases [28]. Although the role of metals in inflammation and inflammation associated diseases, such as OA, is well established, the exact epigenetic mechanisms that underlie their activities are not fully explored. This is the first study to report a full profile of plasma metals in a Greek OA population. No differences were observed between males and females. Compared to other studies in non-Greek OA patients, we report similar levels in circulating Se and Cu [29, 30], but higher levels of Fe and Zn [30] and lower of Cd and Mn [31].

Herein, the sampling strategy selected assured a qualitative data collection. Thus, the thirty-four OA patients had moderate to severe symptoms according to K&L, VAS, and WOMAC, and were mostly non-smokers. The descriptive characteristics of our population coincided with those of the Greek epidemiological study on OA (ESORDIG) reporting higher prevalence of OA in females, aged > 50 years, in obese and non-smokers [32].

Several significant correlations of plasma metals with anthropometrics, disease severity, biochemical, inflammatory/OS markers, QoL and miRNAs were found in our study population.

WOMAC stiffness was positively correlated with Cu and WOMAC total score with Ba. Disease severity has been previously reported to correlate with Cu [29], but, so far, no data exist for its potential relation with Ba levels. Cd, Cs, Ba, and Tl were positively correlated with various dimensions of SF-36 that reflect QoL in OA, although previous studies have shown that exposure to Cd, Cs, and Tl increases OA risk, possibly mediated by biological aging [33]. This paradox finding in our study may in part be explained by the fact that the observed positive correlation between these metals and QoL was not confirmed when adjusting for possible confounders, such as age. Regarding correlations with biochemical and OS markers, our findings confirm already known relations between trace elements and biochemical profile, however, most of them are shown herewith for the first time in Greek OA patients.

Similar to our finding, it has been previously demonstrated that Mn is negatively correlated with HDL in aging men with metabolic syndrome [34]. As expected, Fe, which is beneficial for kidney function was negatively correlated with urea [35] and Rb which is considered nephrotoxic was positively correlated with creatinine [36]. Also, a positive correlation was found between Ni and oxLDL confirming the existing knowledge that Ni exposure is associated with increased OS [37]. In our study Zn was negatively correlated with HDL. Zn supplementation is known to increase HDL levels [38]. Additionally, As was negatively correlated with vitamin D in accordance with recent literature that reports that the effect of As exposure on Th17 function is modified by vitamin D status [39], supporting the negative correlation of As with vitamin D herein. Finally, some studies show that Rb is positively associated with diabetes risk or glucose levels, while others demonstrate the opposite [40–42].

Furthermore, we exhibited some noteworthy correlations of plasma metals with miRNA levels that help explain the complex relationships between lifestyle, environment, and molecular traits of OA. Co correlated positively with miR-21 and mir-146a and Zn inversely with mir-155. These three miRNAs contribute to the pathogenesis of OA and are usually upregulated in the tissues of OA patients [43, 44]. They are already known to regulate inflammatory responses, apoptosis, chondrogenesis and cartilage homeostasis and target key signaling mediators [45, 46]. For example, miR-21 downregulates programmed cell death 4 protein and growth differentiation factor 5 (GDF-5) impairing osteoclastogenesis and chondrogenesis [47], miR-146a upregulates Vascular endothelial growth factor (VEGF) and downregulates Smad4 expression increasing apoptosis rate in chondrocytes [48] and miR-155 inhibits gene expression of autophagy regulators such as Unc-51-like kinase 1 (Ulk1) and Forkhead transcription factor (FoxO3) [49].

Upregulation of miR-21 by Co has been described previously when treatment of human renal epithelial cells with cobalt chloride increased miR-21 levels [50]. A direct association of Co with miR-146a levels has not been shown before, but miR-146a is known to be implicated in the detrimental effects of several metals, such as Cd and Pb and its dysregulation mediates disease progression attributed to heavy metals toxicity [17]. Finally zinc finger proteins are known as direct targets of miR-155 [51], which may explain the negative correlation we found between plasma Zn and miR-155 levels.

After controlling disease activity, age, and gender, a relationship between BMI and IL-6 was present. IL-6 is produced by adipose tissue and the mean values for WC and WHR herewith are indicative of abdominal obesity. According to previous study, obesity increases the crosstalk between chondrocytes and synovial fibroblasts by elevating levels of the pro-inflammatory adipokine leptin, which causes OA patients to produce more IL-6 [52]. Yet, the aforementioned association did not survive when applying the regression model 3. On the contrary, the association between smoking status, as non-smokers vs smokers, and IL-6 survived all three regression models in our study. Knee OA has been shown to have an inverse relationship with smoking [53]. Given that BMI is a key risk factor for OA, there is some evidence to suggest that non-smokers often have higher BMIs than smokers, which results in a high prevalence of OA [54]. However, this needs to be further investigated as a recent observation in the US NHANES 1999–2018 study supports that there is a positive association between smoking and OA prevalence, yet with the major limitations of no physical examination to determine whether participants had OA and the absence of covariates, such as physical activity [55].

When adjusting for disease severity, age, gender, BMI, physical activity level, smoking, TKA and dietary intake of the respective metal (regression model 3), Cu was associated positively with inflammatory markers (CRP, IL-6, and Cu/Zn negatively with WHR. Although weak, the associations of Cu with inflammatory markers herein can be considered as preliminary evidence of a potential in-between link that needs to be further investigated. The role of Cu in OA is well established, being essential for bone health. It promotes regeneration of articular cartilage, enhances chondrogenesis differentiation and regulates cellular and humoral immunity [56]. However, its role may be considered bidirectional; in OA patients, serum Cu levels are higher compared with healthy controls [57] and Cu accumulation in Wilson's disease is related to early-onset of OA [58], probably due to the copper-mediated oxidations and collagen degradation when free Cu occurs in excess [59]. Contradictory findings have been found in the literature about changes in circulation levels with aging; unaltered levels with aging were seen in patients aged 75 to 85 as well as younger subjects (55 to 64 years) [60]. The concentration of blood Cu was higher in healthy older people sampled in Italy than in younger individuals [61]. In our study, plasma Cu levels in patients with moderate to severe OA were comparable with Cu levels in Greek healthy and not-OA adults when assessed applying the exact same methodology [62]. Hereby, in our cohort of OA older-adults, Cu levels were associated with several inflammatory markers (CRP, IL-6) even after adjusting for possible confounders (including Cu dietary intake and TKA) enhancing the robustness of our results. Although Cu’s role may be very important in early stages of life, as it is essential for iron metabolism, immune function, energy production, and neurological system, during aging, when all metabolic functions change, its role may be connected to several diseases [63]. Interestingly, Zhou et al. [64] showed that genetically predicted higher Cu levels are associated with increased risk for OA.

Additionally, we calculated the Cu/Zn ratio, which is suggested as a better biomarker of inflammatory or nutritional changes than Cu and has been associated with several diseases and mortality in elderly [65–67] and correlates with OA severity [31]. Cu/Zn was negatively associated with WHR in our OA patients, independently of their disease severity, age, gender, smoking status, TKA, physical activity and nutritional intakes of Cu and Zn. Cu, Zn or Cu/Zn have been independently associated with BMI and obesity, with Cu and Cu/Zn showing a positive and Zn a negative association [67, 68]. The study of Kerkadi et al. [69] showed the same paradox in a general population, where Zn/Cu ratio positively correlated with waist circumference, although it negatively correlated with BMI and Cu levels positively with BMI.

This is the first study that, to the best of our knowledge, analyzed the whole profile of plasma metals in Greek knee OA patients and assessed how they are connected to several disease-associated variables of the population, including miRNAs. Yet, it displays both strengths and limitations. All questionnaires and tools used in this study (disease severity, QoL, nutritional analysis) are validated and methodologies for metals, miRNAs and biomarkers assessment are well established offering high sensitivity and specificity. However, the small sample size, and the lack of a control sample do not allow us to extrapolate our findings.

## Conclusion

A positive association between plasma Cu levels and inflammatory biomarkers in OA was demonstrated for the first time in the present study. Correlations were pointed between circulating metals and both disease related parameters and miRNAs, partly explaining the role of metals in OA progression. Although not all correlations were confirmed when adjusting for potential confounders, several are reported for the first time in OA patients. Our findings encourage further research on metals related to OA in bigger samples, which will enable us to validate the above correlations and explain the absence of others.

## Data Availability

Upon reasonable request.
